# Population genetics and genome‐wide association studies provide insights into the influence of selective breeding on genetic variation in lettuce

**DOI:** 10.1002/tpg2.20086

**Published:** 2021-02-24

**Authors:** Sunchung Park, Pawan Kumar, Ainong Shi, Beiquan Mou

**Affiliations:** ^1^ USDA–Agricultural Research Service Crop Improvement and Protection Research Unit Salinas CA 93905 USA; ^2^ Department of Horticulture University of Arkansas Fayetteville AR 72701 USA

## Abstract

Genetic diversity is an important resource in crop breeding to improve cultivars with desirable traits. Selective breeding can lead to a reduction of genetic diversity. However, our understanding on this subject remains limited in lettuce (*Lactuca sativa* L.). Genotyping‐by‐sequencing (GBS) can provide a reduced version of the genome as a cost‐effective method to identify genetic variants across the genome. We genotyped a diverse set of 441 lettuce accessions using the GBS method. Phylogenetic and population genetic analyses indicated substantial genetic divergence among four horticultural types of lettuce: butterhead, crisphead, leaf, and romaine. Genetic‐diversity estimates between and within the four types indicated that the crisphead type was the most differentiated from other types, whereas its population was the most homogenous with the slowest linkage disequilibrium (LD) decay among the four types. These results suggested that crisphead lettuces had relatively less genetic variation across the genome as well as low gene flow from other types. We identified putative selective sweep regions that showed low genetic variation in the crisphead type. Genome‐wide association study (GWAS) and quantitative trait loci (QTL) analyses provided evidence that these genomic regions were, in part, associated with delayed bolting, implicating the positive selection of delayed bolting in reducing variation. Our findings enhance the current understanding of genetic diversity and the impacts of selective breeding on patterning genetic variation in lettuce.

## Core Ideas

- Population genetic analysis revealed genetic variation among lettuce horticultural types
- Crisphead lettuce type was the most homogenous than any horticultural types
- GWAS and QTL analyses detected significant association of bolting trait with selective sweeps
- This study provides an understanding of the role of selective breeding in genetic diversity

**Abbreviations**
EHHS: site‐specific extended haplotype homozygosity
*F*_ST_
: fixation index
GBS: genotyping‐by‐sequencing
GWAS: genome‐wide association study
*H*_e_
: heterozygosity
LD: linkage disequilibrium
LOD: logarithm of the odds
QTL: quantitative trait loci
RIL: recombinant inbred line
PCA: principal component analysis
Rsb: ratio of EHHS between populations
SAF: shared allele frequency
SNP: single nucleotide polymorphism.

## 1 INTRODUCTION

Lettuce (*Lactuca sativa* L.) is an important vegetable crop, providing many health benefits such as digestive fibers, phenolic compounds, vitamins, and minerals (Mou, 2008). Lettuce belongs to the Asteraceae with a chromosome number of 2*n* = 18 and its whole‐genome assembly has been recently released (Reyes‐Chin‐Wo et al., 2017). The genus *Lactuca* consists of over 100 species, of which, more than 20 are present in the lettuce gene pool (van Treuren, Coquin, & Lohwasser, 2012) with *L. sativa* and *L. serriola* L. being the main species of the primary gene pool. It is widely accepted that cultivated lettuce has been domesticated from its wild relative *L. serriola* in the Mediterranean region (Harlan, 1992; Kesseli, Ochoa, & Michelmore, 1991), and evidence of its cultivation can be dated back to 2,500 B.C. in Egypt (Lindqvist, 1960). A few other species in the lettuce gene pool, including narrow‐leaf lettuce (*L. saligna* L.) and bitter lettuce (*L. virosa* L.), are also believed to have played a role in the domestication of lettuce (Koopman, Guetta, Wiel, Vosman, & Berg, 1998; Zohary, 1991). The wild relatives continue to be exploited as a source of novel genetic variation in lettuce breeding (Lebeda, Dolezalová, Feráková, & Astley, 2004; Mou, 2008). Thus, a high level of genetic diversity in cultivated lettuce can be attributed to its polyphyletic origin and complex process of domestication (Kesseli et al., 1991; Lindqvist, 1960).

Genetic diversity in the lettuce gene pool is of great importance for the development of improved varieties by providing novel traits and beneficial alleles (Fu, 2015). Through domestication and breeding programs, diverse lettuce varieties with desired traits such as larger leaves and heads, better taste and texture, and lower latex content have been developed (de Vries, 1997; Hartman, Hooftman, Schranz, & van Tienderen, 2013; Mou, 2008). To take full advantage of genetic diversity and help breeders further develop new varieties with improved yield and quality, it is vital to understand the genetic basis underlying the desired traits. For example, delayed bolting is an important trait in lettuce, as premature bolting can lead to bitter flavors and substantial yield loss (Holmes, Wells, Pickens, & Kemble, 2019; Yoshida, Takada, & Koda, 2010). Moreover, bolting is largely accelerated by high temperatures (Al‐Said, 2018; Chen et al., 2018), which will worsen under climate change. Thus, understanding the genetic architecture underlying this trait can facilitate introgression of novel alleles required for sustainable lettuce production under a changing climate.

Based mostly on morphological characteristics, lettuce accessions can be classified into five major horticultural types: butterhead, crisphead (iceberg), leaf, romaine (cos), and stem. These types display considerable differences in several traits including growth rate, leaf texture, size, and shape. Several studies have evaluated the genome‐wide genetic diversity among these types (Kwon, Simko, Hellier, Mou, & Hu, 2013; Simko & Hu, 2008; Zhang et al., 2017) and have concluded that each lettuce type was genetically distinct forming an independent group in a phylogenetic tree. Furthermore, Zhang et al. (2017) identified the genomic regions with reduced genetic variation as putative selective sweeps and found that the regions were specific to each horticultural type. Given that the selective sweeps likely resulted from strong positive selection, their finding suggests that different selective pressures may have been placed on for the different horticultural types. Understanding of the practices associated with selective sweeps will be useful to determine genetic basis of the traits under selection and facilitate introgression of novel alleles. However, our current understanding is limited, warranting further investigations.

Single nucleotide polymorphisms (SNPs), the most common sequence variations encountered in a genome, have been widely used to study the population structure and genetic diversity of germplasm collections (Edwards, Forster, Chagné, & Batley, 2007; Sthapit Kandel, Peng, Hayes, Mou, & Simko, 2020). Single nucleotide polymorphisms have also been used in breeding applications, such as genome‐wide association studies (GWAS) and genomic selection that require a large number of molecular markers covering an entire genome (Truong et al., 2012). Although the cost of sequencing has been reduced substantially with the advent of next‐generation sequencing technologies (Mardis, 2011), the relatively high cost per sample limits their application in breeding. Genotyping‐by‐sequencing (GBS) has been developed as a high‐throughput and cost‐effective alternative for genome‐wide SNP discovery (Poland & Rife, 2012) and has been applied successfully to analyze plant species with large genomes such as lettuce (He et al., 2014; Sthapit Kandel et al., 2020).

**Core Ideas**

- Population genetic analysis revealed genetic variation among lettuce horticultural types
- Crisphead lettuce type was the most homogenous than any horticultural types
- GWAS and QTL analyses detected significant association of bolting trait with selective sweeps
- This study provides an understanding of the role of selective breeding in genetic diversity

In this study, we aimed to assess genetic diversity and elucidate the role of artificial selection in shaping genetic variation in lettuce germplasm. A diverse panel of *Lactuca* spp. accessions was genotyped by GBS. By using the GBS‐derived SNP data, the population structure and phylogenetic relationships among the accessions were studied, and genetic diversity among and within lettuce horticultural types was evaluated. Our results indicated that the crisphead type was most differentiated, and its accessions were more homogenous than those of the other horticultural types. To understand a possible role of positive selection on the reduced genetic diversity in crisphead lettuce, we explored genomic regions under positive selection (i.e., potential selective sweeps) and investigated the bolting trait for its association with the selective sweeps through GWAS and quantitative trait loci (QTL) analyses. The results in this study will improve our understanding of genetic diversity among lettuce germplasm accessions and facilitate the more effective use of diverse germplasm in future breeding programs.

## 2 MATERIALS AND METHODS

### 2.1 Plant materials

A diverse panel of 441 *Lactuca* spp. accessions was selected from the germplasm collections at the USDA–ARS, Salinas, CA, including nine wild lettuce (*L. serriola*) and 432 cultivated accessions (Supplemental Table S1). From this diverse panel, 136 accessions were randomly selected for phenotypic evaluation of bolting time and were grown in a USDA–ARS greenhouse in Salinas, CA. An interspecific recombinant inbred line (RIL) population (152 lines) derived from a cross between ‘Salinas’ (crisphead) and US96UC23 (*L. serriola*) (Truco et al., 2013) was also evaluated for bolting time in the same greenhouse. The plants were grown on potting mix soil (Sun Land Garden Products) in 6.4‐cm pots under natural light conditions at a mean air temperature between 17 and 33 °C.

### 2.2 SNP identification and imputation

Leaf tissues of all accessions were submitted to Data2Bio (Ames, IA) for DNA extraction and genotyping. Genomic sequencing and SNP calling were conducted using tunable GBS technology (Ott et al., 2017). Briefly, sequence reads were aligned to a reference genome sequence of cultivar Salinas (Reyes‐Chin‐Wo et al., 2017) using GSNAP software (Wu & Nacu, 2010) and only reads with a single unique alignment were selected. Single nucleotide polymorphisms were initially filtered using the following criteria: (a) PHRED base quality ≥ 20; (b) biallelic; (c) for homozygous genotypes, SNPs were supported by ≥5 reads and ≥90% of all aligned reads; (d) for heterozygous genotypes, the reference and alternative alleles were supported by ≥2 reads and ≥20% of the reads.; and (e) sites that failed to pass the above criteria were assigned as missing genotypes.

The initial set of SNPs was further filtered: the samples with missing SNP genotypes of >20% were excluded; SNPs with missing rate of >20% and minor allele frequency of <5% were excluded using the vcftools program (Danecek et al., 2011). The final, filtered set of SNPs was subsequently used for imputations. The Beagle v5.0 (Browning, Zhou, & Browning, 2018) that is based on the localized haplotype clustering was used to impute missing genotypes. In order to achieve high imputation accuracy, an empirical approach was taken: (a) 1% of genotypes across samples for each SNP site were randomly masked as missing to measure imputation accuracy; (b) the imputation was done using the Beagle software with default parameters, except for iterations = 30; (c) imputation accuracy was evaluated as a ratio of correctly imputed genotypes to total masked genotypes, and only SNP sites with at least 95% accuracy rate were selected. Finally, a single variant call format file with 186,008 SNPs was generated for downstream analyses. The SNP annotation (i.e., synonymous or nonsynonymous) based on the reference protein coding sequences and transition/transversion ratio analysis were conducted using custom perl scripts.

### 2.3 Population genetic analysis

The R‐package poppr (Kamvar, Tabima, & Grünwald, 2014) was used to construct a phylogenetic tree based on the imputed SNP data using the neighbor‐joining method in which *L. serriola* was used as an outgroup (Saitou & Nei, 1987). The pairwise genetic distances between the *Lactuca* accessions were estimated using the bitwise.dist method that measures the Hamming distance (Anselmo & Pinheiro, 2012) and were used to construct a dendrogram for which bootstrap replicates were set to 1,000. The final tree was visualized using the FigTree v1.4.3 (http://tree.bio.ed.ac.uk/software/figtree).

Principal component analysis (PCA) and LD analysis were performed using the PLINK version2.0 (Chang et al., 2015). The first two principal components for the lettuce accessions were plotted using the R package ggplot2 (R Core Team, 2019). Linkage disequilibrium (*r* ^2^) was calculated for all pairs of SNPs using PLINK with following parameters: –r2, –ld‐window‐r2 0, –ld‐window 999999, and –ld‐window‐kb 5000. The *r* ^2^ values were averaged into 10‐kb distance bins and the average values were plotted against the mid‐distance of each bin.

The fastSTRUCTURE program version 2 (Raj, Stephens, & Pritchard, 2014) was used to infer population structure. The optimal number of model components (*K*) was determined by a function of chooseK.py based on admixture proportions (*Q* values), and *Q* values in each accession were estimated for a given population number from *K* = 2 to *K* = 4. Ancestry and admixture proportions were visualized using R‐graphic tools (R Core Team, 2019).

### 2.4 Genetic diversity within and between lettuce horticultural types

To explore the genetic divergence between populations of horticultural types, pairwise fixation index (*F*_ST_) values between the four types were calculated using the method of Weir and Hill (2002) implemented in the R package BEDASSLE (Bradburd, 2014). The fixation index is a measure of the allele frequency variation between populations, ranging between 0 and 1, with higher *F*_ST_ implying greater differentiation. Genetic diversity within population was measured by two methods: (a) an average of expected heterozygosity for all loci in a population (Nei, 1973) and (b) frequency of shared alleles between all individuals in a population (Chakraborty & Jin, 1993).

### 2.5 Positive selection analysis

To detect genomic regions under positive selection in the crisphead type, site‐specific extended haplotype homozygosity (EHHS) and the ratio of EHHS between populations (Rsb) were estimated using the R‐package reh’ (Gautier, Klassmann, & Vitalis, 2017). First, EHHS values per SNP marker were estimated for a group of crisphead accessions and for the remaining population, which served as a reference. The EHHS is the probability that any two randomly chosen chromosomes from a population are homozygous over the given surrounding chromosomal region of a focal SNP marker. Next, the Rsb value (a standardized ratio of the EHHS values from comparing two populations) was computed for each SNP site. The regions with average Rsb value of >3 and *P* value < .001 within sliding windows of 1 Mb (step size 100 kb) were selected, and the regions, if overlapped, were merged and determined as putative selective sweeps (Supplemental Table S2)

### 2.6 Phenotyping, GWAS, and QTL analysis

To measure bolting time, three replicates of each individual line were planted in a greenhouse in a randomized complete‐block design as described in the Plant Materials section above. The bolting time of lettuce plants was scored as the number of days from sowing to bolting, with bolting defined as when the main stem elongated to 10 cm.

For GWAS, lettuce accessions (*n* = 136), including five *L. serriola* accessions, were sown in July 2019 (see Supplemental Table S3 for phenotypes). Association analysis was conducted using the R‐package Genome Association and Prediction Integrated Tool (GAPIT) version 3 (Lipka et al., 2012) with the integrated method, Settlement of MLM Under Progressively Exclusive Relationship (SUPER) (Wang, Tian, Pan, Buckler, & Zhang, 2014). Population stratification was corrected by PCA. The optimal number of principal components was determined by a Bayesian information criterion‐based analysis in GAPIT. Association analysis in GAPIT was performed with parameters PCA.total = 3 and model = SUPER while leaving all other parameters at their default settings. Significant association of SNPs with bolting time was determined with a threshold of false discovery rate equal to 0.05, which is equivalent to −log_10_ (*P* value) > 4.44 (Supplemental Table S4).

For QTL mapping of bolting time, 152 RILs were sown in August 2019 that had been genotyped with lettuce 6.6 million feature Affymetrix high density GeneChip at the University of California, Davis, CA (https://michelmorelab.ucdavis.edu). The mean values of days to bolting were Box–Cox transformed to normalize trait values (Box & Cox, 1964) and analyzed using the R‐package qtl (Broman, Wu, Sen, & Churchill, 2003). The threshold for a significant logarithm of the odds (LOD) value for QTL was determined by 1,000 permutations of the phenotypic data. The best multiple‐QTL model was determined using Haley–Knott regression and the stepwise model selection procedure (stepwiseqtl) (Manichaikul, Moon, Sen, Yandell, & Broman, 2009). We used ANOVA (fitqtl) to calculate the LOD score and percentage variation explained for each QTL and then refitted the model to generate effect sizes. The genes located within a Bayesian 95% confidence interval for a QTL were annotated with Gene Ontology assignments as described by Park, Shi, and Mou (2020).

## 3 RESULTS

### 3.1 Distribution and properties of SNPs in the lettuce genome

We selected 441 *Lactuca* accessions for GBS‐based genotyping, including nine accessions of wild lettuce (*L. serriola*) and 432 lettuce accessions (126 butterhead, 104 crisphead, 92 leaf, 102 romaine, and eight stem lettuce). After filtering the raw GBS sequencing data and filling in missing genotypes through imputation, a total of 186,008 SNPs were obtained. The density of SNP markers per megabase ranged from 53 SNPs on chromosome 6 to 98 SNPs on chromosome 1, with an average density of 81 SNPs across the whole genome (Supplemental Figure S1). Of all SNPs, 98,653 SNPs (53%) displayed a minor allele frequency of 15% or less, while 122,008 SNPs (66%) had a heterozygosity rate of 10% or less (Supplemental Figure S2).

**FIGURE 1:**
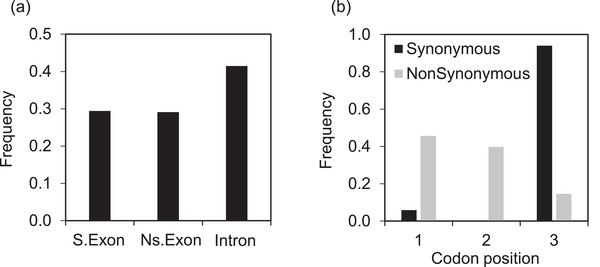
Distribution of single nucleotide polymorphisms (SNPs) in genic region of the lettuce genome. (a) Frequency of synonymous (S.Exon) and nonsynonymous (Ns.Exon) SNPs in exon and SNPs in intron based on the gene models in the lettuce reference genome. (b) Frequency of synonymous and nonsynonymous SNPs at the three codon positions

We annotated the SNP loci based on predicted gene models from the lettuce reference genome to evaluate their effects on the protein sequences. Most of SNP loci were located within intergenic regions, and only 0.94% (*n* = 1,746) of SNP loci were within genic regions (Figure 1; Supplemental Table S5). Of the genic SNP loci, 41% (*n* = 724) were located in intron regions, and 59% (*n* = 1,022) were located in exon regions. About half (*n* = 508) of the exonic SNPs were predicted to result in a change in the amino acid (i.e., nonsynonymous), and the other half (*n* = 514) represented synonymous substitutions (Figure 1a). If the probability of all possible substitutions is equal, 76% of substitutions are expected to be nonsynonymous (Wilke, 2004). As such, our observed nonsynonymous rate indicated that there was strong selection pressure against nonsynonymous changes in the genes studied. Codon positions also influence the type of substitutions as they have different functional constraints to mutations (Bofkin & Goldman, 2007). We examined the positions of the synonymous and nonsynonymous SNPs in the codon. As expected, because of codon degeneracy, nearly all of the synonymous SNPs (94%) were located in the third position, followed by 6% in the first position, and only 0.2% in the second position; whereas, the nonsynonymous SNPs were located mostly in the first (46%) or second (40%) positions followed by 14% in the third position (Figure 1b).

Next, we estimated the ratio of transition to transversion for all SNPs. The transition SNPs (67.8%) were found higher than the transversion SNPs (32.2%), resulting in the transition/transversion ratio of 2.1. When considering SNPs within genic regions, transitions and transversions were 62.8 and 37.2%, respectively, resulting in the transition/transversion ratio of 1.68. This ratio agrees with those based on transcriptome in kok‐saghyz (*Taraxacum kok‐saghyz* L. E. Rodin) with 1.65 (Luo, Iaffaldano, Zhuang, Fresnedo‐Ramírez, & Cornish, 2017), rubber tree [*Hevea brasiliensis* (Willd. ex A. Juss.) Müll. Arg.] with 1.5 (Mantello et al., 2014), and chicory (*Cichorium intybus* L.) with 1.61 (Galla, Ghedina, Tiozzo, & Barcaccia, 2016).

### 3.2 Population structure and genetic relationships

**FIGURE 2:**
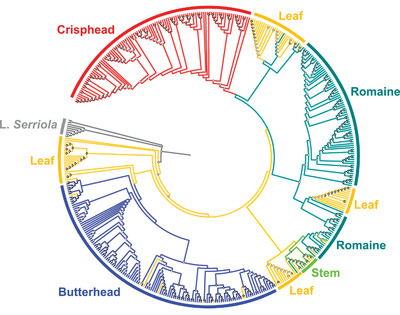
Phylogenetic tree of wild and cultivated lettuce. The tree was constructed based on 186,008 single nucleotide polymorphisms of nine *L. serriola* and 432 lettuce accessions using the neighbor‐joining method with *L. serriola* as an outgroup. Colors indicate horticultural types and *L. serriola* (red, crisphead; gold, leaf; cyan, romaine; blue, butterhead; green, stem; grey, *L. serriola*). Open diamonds on nodes indicate bootstrap support greater than 50%

To determine evolutionary relationships among the 441 *Lactuca* accessions, a phylogenetic tree was constructed using the neighbor‐joining method based on the GBS‐derived SNP genotypes (Figure 2). In the phylogenetic tree, the leaf type appeared to be most closely related to *L. serriola*, followed by the butterhead and romaine types, and crisphead was most distantly related. Furthermore, lettuce accessions were categorized into distinct subclades by their horticultural types with a few exceptions. For example, leaf‐type accessions were located between or within subclades of other types and the stem types were mostly clustered with the leaf type.

**FIGURE 3:**
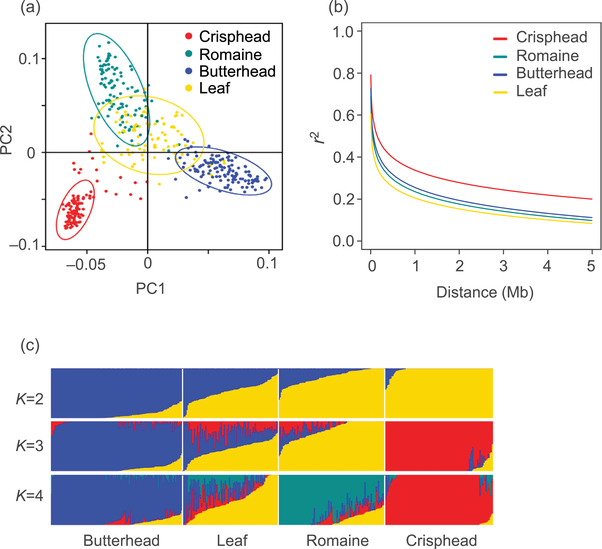
Population genetic analyses of 424 accessions from four horticultural types of lettuce. Colors indicate horticultural types: red, crisphead; gold, leaf; cyan, romaine; blue, butterhead. (a) Principal component (PC) analysis plot of the lettuce accessions. Ellipses indicate 95% confidence interval. (b) Linkage disequilibrium (LD) decay measured by a pairwise LD values (*r* ^2^). Nonlinear logarithmic regression curves of *r* ^2^ are plotted against genetic distance (expressed in Mb). (c) Population structure plot with different numbers of clusters (*K* = 2–4), based on ancestry probabilities for the 424 accessions estimated from a fastSTRUCTURE analysis. The *x* axis shows the different horticultural types

To understand the genetic structure of four of the main horticultural types, we conducted PCA with accessions from butterhead, crisphead, leaf, and romaine types. Stem type and *L. serriola* were excluded to prevent potential biases in representing those groups because of their relatively small numbers of accessions. The first two principal components explained 51% of the total variation with the first and second components explaining 33 and 18%, respectively. As observed earlier in the phylogenic analysis, accessions were largely structured according to their horticultural types in the PCA plot. However, considerable overlap was observed between the leaf, romaine, and butterhead types (Figure 3a). The accessions of leaf type were located in the center and overlapped mostly with romaine type and to some extent with butterhead type, indicating genetic closeness between these types. In contrast, crisphead was well separated from all other types showing no overlap with any other types at the 95% confidence limit in the PCA plot.

We also estimated LD as the correlation coefficient (*r* ^2^) between the 186,008 SNPs for the four horticultural types, and the average *r* ^2^ values within 10‐kb intervals were plotted against physical distance between SNP pairs (Figure 3b). Linkage disequilibrium declined rapidly with increasing distance between SNP markers, and this rate of decline varied between horticultural types. The LD decay was measured as the physical distance at which LD decreases to half of its maximum value. Among the four horticultural types, leaf type showed the shortest LD decay of 241 kb (*r* ^2^ = .313), followed by romaine with 349 kb (*r* ^2^ = .340), and butterhead with 388 kb *(r* ^2^ = .339). The crisphead type exhibited the longest LD decay of 746 kb (*r* ^2^ = .363).

Population structure was analyzed with the same SNP data set using the fastSTRUCTURE program (Figure 3c). The number of model components (*K*) explaining the best population structure was estimated to be two. However, to examine the extent to which lettuce types were admixed with each other, we applied *K* = 2–4. At *K* = 2, butterhead type appeared as a subgroup and the other types were assigned to the second group. At *K* = 3, crisphead type appeared to be a distinct subgroup, and at *K* = 4, the romaine and leaf type were separated into two subgroups. Despite each lettuce type largely assigned to a structural group, they exhibited varied levels of admixture proportions. Butterhead and crisphead showed relatively lower admixture proportions than the romaine type, whereas the leaf‐type lettuce showed the highest levels of admixture. The higher admixture proportions in the leaf type were in agreement with the PCA result in which leaf type overlapped most with the other types. In addition, the lower admixture proportions of crisphead type were consistent with the lack of overlap observed in the PCA plot, indicative of low gene flow.

The extent of genetic differentiation between the horticultural types was estimated using pairwise *F*_ST_ values, where higher *F*_ST_ indicates greater genetic differentiation (Weir & Hill, 2002). Overall, the pairwise *F*_ST_ values ranged from .106 to .357 with a mean value of .219 (Table 1). The lowest *F*_ST_ value was observed between leaf and romaine, the two types showing the most overlap in the PCA plot, whereas crisphead and butterhead showed the highest *F*_ST_ value, suggesting a distant relationship between the two types. Furthermore, crisphead showed the highest average value of .275, followed by butterhead with .248, romaine with .206, and leaf with .149. Taken together with PCA and population structure analysis, these results supported that the crisphead type is most differentiated of the four types.

**TABLE 1:** Pairwise fixation index (*F*_ST_) values between four lettuce types based on single nucleotide polymorphism genotypes

	Butterhead	Crisphead	Leaf	Romain
Butterhead	0	–	–	–
Crisphead	.357	0	–	–
Leaf	.138	.204	0	–
Romain	.249	.265	.106	0

*Note*. Significance tests were performed using 1000 permutations. All values are statistically significant (*P* < .001).

The extent of genetic diversity within each horticultural type was estimated as expected heterozygosity (*H*_e_) (Nei, 1973) and shared allele frequency (SAF), where higher *H*_e_ or lower SAF indicates greater diversity within a population (Table 2). The diversity values indicated that leaf type was most genetically diverse with the highest *H*_e_ (0.27) and lowest SAF (0.72) values. The butterhead and romaine had intermediate diversity with *H*_e_ (0.23 and 0.22, respectively) and SAF (0.78 and 0.79, respectively). The lowest diversity was observed in the crisphead type with the lowest *H*_e_ (0.13) and highest SAF (0.87). Thus, the crisphead types were the most homogeneous of the four horticultural types.

**TABLE 2:** Expected heterozygosity (*H*_e_) and shared allele frequency (SAF) within a type

	*H*_e_	SAF
Butterhead	0.23	0.78
Crisphead	0.13	0.87
Leaf	0.27	0.72
Romaine	0.22	0.79

### 3.3 Identification of genomic regions under positive selection in the crisphead type

**FIGURE 4:**
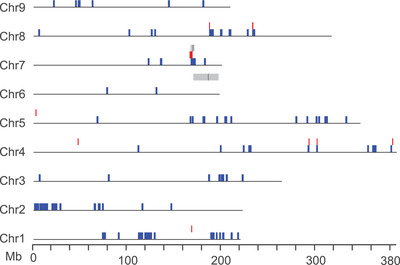
Distribution of potential selective sweeps of crisphead, and genome‐wide association study (GWAS) and quantitative trait loci (QTL) for bolting time across the lettuce genome. Potential selective sweeps (blue bars) with low genetic variation in the crisphead type were compared with GWAS (red bars) and QTL (grey boxes) for bolting time. The length of grey boxes represents a 95% confidence interval for the QTL and black bars within the grey box represent the QTL peak. Scale bar at the bottom indicates genetic distance (Mb)

In general, selective breeding (i.e., artificial selection) causes a new beneficial allele to be fixed rapidly with increased frequency in the population, which can lead to the reduction or elimination of genetic variation over the desired allele and nearby regions. Such rapid fixation of beneficial alleles is called a selective sweep (Turner‐Hissong, Mabry, Beissinger, Ross‐Ibarra, & Pires, 2020; Yamasaki, Wright, & McMullen, 2007). Given the lowest genetic diversity as well as the highest LD decay in the crisphead type, we questioned whether artificial selection influenced its patterns of genetic variation. To address this question, we first searched for relatively conserved (i.e., low genetic variation) regions in a population of crisphead as selective sweeps in comparison with the other types using genome‐wide SNP data. We detected a total of 72 potential selective sweeps for the crisphead type with an average length of 2.5 Mb. The regions comprised ∼8% (185 Mb) of the lettuce genome and contained 3,020 genes (7.8%). (Figure 4, Supplemental Table S2).

**FIGURE 5:**
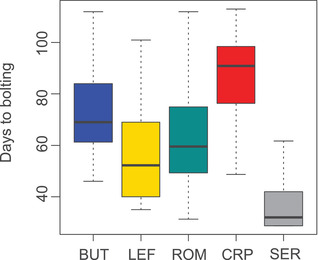
Bolting time for four lettuce horticultural types and *L. serriola*. Bolting time was measured as the number of days from sowing to bolting for butterhead (BUT, *n* = 35), leaf (LEF, *n* = 31), romaine (ROM, *n* = 36), crisphead (CRP, *n* = 29), and *L. serriola* (SER, *n* = 5)

**FIGURE 6:**
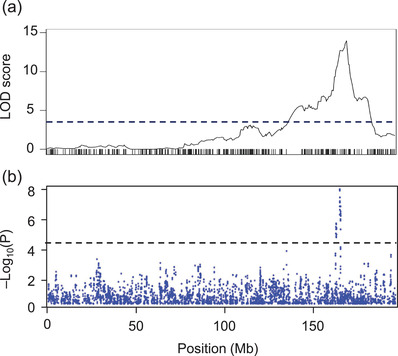
Genomic region in chromosome 7 contributing to delayed bolting in lettuce identified by (a) quantitative trait loci (QTL) and (b) genome‐wide association mapping. (a) Tick marks on the *x* axis represents genetic markers used for QTL mapping and the dashed line represents logarithm of odds (LOD) threshold obtained with 1000 permutation tests (*P* = .01) (b) The dots in the Manhattan plot represent single nucleotide polymorphism markers. The dashed line corresponds to the genome‐wide false discovery rate (FDR = .05) threshold. The *x* axis represents physical distance (Mb) of chromosome 7

Selective sweeps are believed to result from strong positive selection for beneficial traits (Turner‐Hissong et al., 2020). Given its importance in lettuce yield, delayed bolting would be one such candidate trait. Our test of bolting time on a subset of the GBS population showed that all cultivated lettuces bolted later than *L. serriola*, and among the four horticultural types, the crisphead type showed the most delayed bolting (Figure 5). Compact head formation is a defining characteristic of crisphead lettuce, and early onset of bolting, where the compact head formation is disrupted, can lead to severe yield loss in this lettuce type. Thus, it is not surprising that the crisphead type displayed the most delayed bolting. We assumed that the selective sweeps in this type might be associated with delayed bolting. To investigate this possibility, we performed the association analysis for bolting time with the accessions randomly selected from the GBS population including 29 crisphead, 35 butterhead, 31 leaf, 36 romaine type, and five *L*. *serriola*. These accessions displayed substantial variation in days to bolting, ranging from 26 to 113 d with an average of 68 d (Supplemental Figure S3). We conducted GWAS for bolting time based on the imputed SNP data. A total of 146 SNPs were significantly associated with bolting time, with a cutoff of FDR = .05 (*P* value < 3.6 × 10^−5^), spanning nine regions across the genome (Supplemental Figure S4, Supplemental Table S4). We compared these regions with the selective sweeps in crisphead identified earlier in this study. Of the nine regions, five (two regions on chromosome 4, one on chromosome 7 and two on chromosome 8) overlapped with the selective sweeps of crisphead, and one region at the distal end of chromosome 4 was located near the selective sweeps (Figure 4). The SNPs most significantly associated were located in a region of ∼164 Mb on chromosome 7 (Figure 6; Supplemental Figure S4).

To validate the GWAS results, we also conducted a biparental QTL analysis using a RIL population (*n* = 152) derived from a cross between a crisphead cultivar (Salinas) and a *L. serriola* accession (US96UC23). A total of 3,261 genetic markers segregating in this population were used to map loci controlling bolting time. Accession US96UC23 bolted at an average of 35 d and Salinas bolted much later at an average of 81 d, whereas the F_1_ hybrid plants bolted at an average of 57 d, close to the parental midpoint (Supplemental Figure S5a). The RIL population showed a right‐skewed distribution in bolting, ranging from 27 to 103 d with an average of 46 d (Supplemental Figure S5b). This phenotypic distribution suggested polygenic or oligogenic control of bolting in *Lactuca* spp. Quantitative trait loci analysis mapped a substantial fraction of this bolting time variation to two genomic regions at chromosome 6 and 7, named BLT6 and BLT7, respectively (Figure 4, Table 3). These two loci showed LOD scores of 3.7 for BLT6 and 14 for BLT7, explaining 7.5 and 33.1% of the total phenotypic variance, respectively (Table 3). For both loci, alleles from Salinas resulted in late bolting. When compared with the GWAS results and selective sweeps, we found that the BLT7 locus overlapped with the genomic region that was most significantly associated in our GWAS analysis and also was detected as potential selective sweep (Figures 4 and 6). Thus, this result supported the idea that genomic regions associated with delayed bolting were under positive selection in crisphead lettuce, resulting in low sequence variation (i.e., selective sweeps).

**TABLE 3:** Quantitative trait loci (QTL) for variation in bolting response

QTL	Chromosome	Position	LOD^a^	Bayesian 95% confidence interval	Effect size	PVE^b^
		cM				
BLT6	6	136.0	3.7	122.9–141.9	5.4	7.5
BLT7	7	122.3	14.0	119.7–122.3	13.5	33.1

*Note*. Effective sizes are quantified as the difference in mean bolting time (days) between genotypes homozygous for the ‘Salinas’ and US96UC23 allele, respectively; Positive values indicate that the ‘Salinas’ genotype is associated with later bolting.

^a^ LOD, logarithm of the odds.

^b^ PVE, percentage variance explained by the QTL.

To determine potential candidate genes responsible for delayed bolting, we examined all 114 genes within the Bayesian 95% confidence interval of the BLT7, which corresponds to genomic regions between markers BIJT (163902772‐bp) and CCJJ (165567585‐bp) in chromosome 7 (https://michelmorelab.ucdavis.edu). We associated these genes with Gene Ontology biological functions and determined genes as potential candidates if associated with bolting‐relevant functions of photoperiodism, circadian, *or reproductive phase. Based on these criteria, five genes were identified: Phytochrome C, CONSTANS*‐like 9, *UV‐B resistance 8* (*UVR8*), *Heterogeneous nuclear ribonucleoprotein 1*, and *STERILE APETALA* (Supplemental Table S6).

## 4 DISCUSSION

Understanding the extent of genetic diversity in crop germplasms is of utmost importance as it forms a basis for selecting parental lines for developing new improved varieties. In this study, we performed genome‐wide surveys of genetic variation in 441 accessions of *Lactuca* spp., using 186,008 SNP markers developed from a method of GBS. The genetic diversity analysis revealed that crisphead lettuce is most distantly related to other horticultural types, while the diversity within the crisphead type is considerably lower than for other horticultural types. In addition, we identified the genomic regions under positive selection in the crisphead type and provided evidence through GWAS and QTL analyses that some of the regions were associated with domestication traits such as delayed bolting.

Substantial phenotypic variation exists among horticultural types of lettuce such as appearance, leaf texture, height, and color. Consistent with the phenotypic variation, our phylogenetic analysis based on genome‐wide SNP markers showed that each type formed a distinct cluster, reflecting strong genetic differentiation among the four types studied. However, a few exceptions were observed. One such exception is that leaf‐type accessions fall into the clusters of other types (Figure 2). This topology of leaf type was also observed by Zhang et al. (2017), where some leaf‐type accessions were dispersed across the clusters of other horticultural types. Both studies provide evidence that there has been considerable gene flow among lettuce horticultural types. Particularly intriguing is that mostly leaf‐type accessions fall into the clusters of other horticultural types, not the other way around. In other words, gene flow appears to occur mostly onto leaf type from other types. Assuming that the distinct morphology of lettuce horticultural types is likely associated with their unique genetic make ups, this observation suggests that the leaf type might be dominant over the other horticultural types in morphological traits. Leaf‐type accessions that are associated with types outside their own group indeed showed the distinct morphology of leaf type (unpublished data). This idea could be tested further by investigating morphological trait loci and alleles specific to leaf‐type lettuce.

A second exception is that the stem type accessions were located with the leaf‐type cluster (Figure 2). This observation indicated that the genetic distance between stem and leaf types was smaller than between outlying leaf accessions, at least for the stem‐type accessions studied. Consistent with this observation is that stem type is believed to have been derived from leaf type (de Vries, 1997). However, Zhang et al. (2017) categorized stem type into an independent monophyletic cluster, closely allied to leaf type in their phylogenetic analysis. The different results would be due to the source of genetic variations. Zhang et al. (2017) estimated genetic distances from transcriptome‐based SNPs, whereas whole‐genome‐based SNPs were used for estimates in our study, with most SNPs located in intergenic regions. Thus, it is possible that estimates might be biased when solely based on the expression SNPs. Another possibility is that genetic variation between stem and leaf types might be greater in the transcriptome than nongenic regions. The answer awaits further analysis based on unbiased comprehensive genetic variation data.

Among the four horticultural types studied, the crisphead type showed the lowest expected heterozygosity and the highest shared allele index, indicating that crisphead type is more homogeneous than any other type. Our findings provided evidence that strong selection pressure for delayed bolting has played a role in limiting genetic variation within crisphead lettuces. We identified relatively conserved genomic regions in crisphead lettuces as putative selective sweeps in comparison with the other types and found that those regions were in part associated with delayed bolting through GWAS and QTL analyses (Figure 4). Together, these results implicate positive selection for delayed bolting in reducing genetic diversity in the crisphead type. However, the effect is likely limited to the regions associated with desired alleles, and the positive selection alone might be insufficient to explain the reduced genetic variation across the genome. In general, crop breeding occurs on a relatively short time scale, thus predominantly relying on existing genetic diversity, and the practice of artificial selection on a limited gene pool can lead to population bottlenecks (i.e., founder effects), which consequently lead to reduced genetic variation (Hyten et al., 2006; Turner‐Hissong et al., 2020). Reduced genetic diversity in the crisphead type may be attributed to this population bottleneck effect. Historical data show that modern American crisphead lettuce was derived in the 20th century from the French Batavia types (de Vries, 1997). Since then, most modern crisphead cultivars have been developed in the United States where, according to Mikel (2007), three cultivars (Calmar, Salinas, and Vanguard) were heavily used as parents. A majority (60%) of crisphead accessions in this study did originate in the United States and 15% were of unknown origin (Supplemental Table S1). Intensive use of a limited number of parental lines and relatively recent development (i.e., low number of generations) may both have contributed to shaping the standing genetic diversity of modern crisphead types.

Genome‐wide and local reduction of genetic diversity may impair the future efficiency of selection in lettuce breeding. Moreover, selective sweeps could increase the fixation probability of weakly deleterious alleles that hitchhike with the selected alleles as a result of linkage drag in the population (Olsen & Wendel, 2013; Varshney et al., 2017). In order to improve response to future selection, the crisphead type may require targeted introgression from secondary and tertiary gene pools and larger population sizes to maximize recombination among elite parental lines.

## AUTHOR CONTRIBUTIONS

Sunchung Park: Conceptualization; Data curation; Formal analysis; Investigation; Methodology; Project administration; Resources; Software; Validation; Visualization; Writing‐original draft; Writing‐review & editing. Pawan Kumar: Investigation; Methodology; Resources; Writing‐review & editing. Ainong Shi: Funding acquisition; Methodology; Resources; Writing‐review & editing. Beiquan Mou: Conceptualization; Funding acquisition; Methodology; Project administration; Resources; Supervision; Writing‐review & editing

## AUTHOR CONTRIBUTIONS

SP and BM conceptualized the study and designed experiments. BM and AS obtained funding for this investigation. SP conducted the experiments and analyzed the results with help from PK, BM, and AS. All authors wrote the manuscript, reading and approving the final version.

## CONFLICT OF INTEREST

The authors declare that the research was conducted in the absence of any commercial or financial relationships that could be construed as a potential conflict of interest.

## Supporting information

Supplemental Figure S1. SNP density with a 10 Mb window across the genome.
Supplemental Figure S2. Distribution of minor frequency allele (MAF) (a) and heterozygosity (b) for SNPs.
Supplemental Figure S3. Frequency distribution of days‐to‐bolting for the 136 lettuce accessions used for GWAS.
Supplemental Figure S4. Manhattan plot of genome‐wide association for days‐to‐bolting (number of accessions = 136).
Supplemental Figure S5. Days‐to‐bolting phenotype for ‘Salinas’, US96UC23, and F1 plants (a) and frequency distribution of days‐to‐bolting among ‘Salinas’ x ‘US96UC23’ RIL population (*n* = 152) (b)

Supplemental Table S1. List of the 441 *Lactuca* spp. accessions used for GBS

Supplemental Table S2. Genomic regions under positive selection in crisphead type lettuce

Supplemental Table S3. Bolting‐time data of the accessions used for GWAS

Supplemental Table S4. List of SNPs significantly (FDR = .05) associated with bolting time based on GWAS analysis.

Supplemental Table S5. List of SNPs located in genic region and their effects on amino acid substitution

Supplemental Table S6. List of candidate genes for bolting QTL on chromosome 7

## Data Availability

The GBS‐based SNP data are available in the European Variation Archive (https://www.ebi.ac.uk/eva) under project number PRJEB40369. All relevant data are included in the manuscript and the supplemental material.
