# Supplementary material for: Population genetics and genome‐wide association studies provide insights into the influence of selective breeding on genetic variation in lettuce
Source: Plant Genome. 2021 Feb 24;14(2):e20086. doi: 10.1002/tpg2.20086 (PMC12807027; doi:10.1002/tpg2.20086)
Supplement: Supplementary file 1 [file TPG2-14-e20086-s001.docx]

**Population genetic and genome-wide association studies using genotyping by sequencing (GBS) provide an insight into the influence of selective breeding on genetic variation in lettuce**

**Sunchung Park^1^, Pawan Kumar^1^, Ainong Shi^2^, and Beiquan Mou^1,^***

**Supplementary information**

Supplemental Figure S1-5

Supplemental Table S1-6

**Supplemental Figure S1. SNP density per Mb with a 10 Mb sliding window across the genome**. The x-axis represents the physical distance along each chromosome (Mb). The y-axis represents the chromosome numbers.

**Supplemental Figure S2. Distribution of minor frequency allele (MAF) (a) and heterozygosity (b) for SNPs.** Frequency is indicated as percentage of the total number of SNPs.

**Supplemental Figure S3. Frequency distribution of days-to-bolting for the 136 lettuce accessions used for GWAS.**

**Supplemental Figure S4. Manhattan plot of genome-wide association mapping for days-to-bolting (number of genotypes =136).** Negative log_10_-transformed *P* values from a genome-wide scan are plotted against position on each of the 9 chromosomes. The red line corresponds to the genome-wide false discovery rate (FDR = 0.05) threshold.

**Supplemental Figure S5. Days-to-bolting phenotype for Salinas, US96UC23, and F1 plants (a) and frequency distribution of days-to-bolting for ‘Salinas’ x ‘US96UC23’ RIL population (n=152) (b).**

**Supplemental Tables**

Supplemental Table S1. List of the 441 *Lactuca* spp. accessions used for GBS

Supplemental Table S2. Genomic regions under positive selection in crisphead type lettuce

Supplemental Table S3. Bolting-time data of the accessions used for GWAS

Supplemental Table S4. List of SNPs significantly (FDR=0.05) associated with bolting based on GWAS analysis

Supplemental Table S5. List of SNPs located in genic region and their effects in amino acid substitution

Supplemental Table S6. List of candidate genes for Bolting QTL on chromosome 7
